# Bioactive C21 Steroidal Glycosides from *Euphorbia kansui* Promoted HepG2 Cell Apoptosis via the Degradation of ATP1A1 and Inhibited Macrophage Polarization under Co-Cultivation

**DOI:** 10.3390/molecules28062830

**Published:** 2023-03-21

**Authors:** Xiaoyi Feng, Jianchun Li, Hongmei Li, Xuanqin Chen, Dan Liu, Rongtao Li

**Affiliations:** 1 Center for Pharmaceutical Sciences and Engineering, Faculty of Life Science and Technology, Kunming University of Science and Technology, Kunming 650500, China; 2 Faculty of basic Medicine, Yunnan University of Chinese Medicine, Kunming 650500, China

**Keywords:** *Euphorbia kansui*, antitumor, immunomodulatory, Na^+^, K^+^-ATPase, α1 subunit of NAK, dual antitumor activity

## Abstract

*Euphorbia kansui* is clinically used for the treatment of esophageal cancer, lung cancer, cancerous melanoma, asthma, pleural disorders, ascites, and pertussis, among other conditions. In this study, 12 steroids were obtained and identified from *E. kansui*, and cynsaccatol L (**5**), which showed the best effects in terms of inhibiting the proliferation of HepG2 cells and the immune regulation of macrophages. Furthermore, **5** induced typical apoptotic characteristics in HepG2 cells, such as morphological changes and the caspase cascade, as well as inducing autophagy-dependent apoptosis via mitochondrial dysfunction and reactive oxygen species (ROS) accumulation. The antitumor mechanism of **5** might be related to promoting the endocytosis and degradation of ATP1A1 protein and then down-regulating the downstream AKT and ERK signaling pathways. Furthermore, the antiproliferation effect of **5** in co-cultivation with macrophages was investigated, which showed that **5** promoted the apoptosis of HepG2 cells by modulating the release of inflammatory cytokines, such as TNF-α and IFN-γ; regulating the M2-subtype polarization of macrophages; promoting the phagocytosis of macrophages. In conclusion, **5** exerted anti-proliferative effects by promoting the degradation of ATP1A1 and inhibiting the ATP1A1-AKT/ERK signaling pathway in HepG2. Furthermore, it regulated macrophage function in co-cultivation, thereby further exerting adjuvant anti-HepG2 activity.

## 1. Introduction

*Euphorbia kansui* is widely distributed in the central plains, mountains, and Sichuan basins in China [1]. It has been used for more than 2000 years, mainly for the treatment of esophageal cancer, lung cancer, cancerous melanoma, chronic bronchitis, acute pancreatitis, asthma, pleural effusions, and ascites, as well as intestinal obstruction and pertussis [2]. Research on the material basis of *E. kansui* shows that its main components are diterpenes and triterpenes [3], which are also its main toxicity components [4,5]. Recently, studies have shown that steroids are also important active ingredients that show extensive bioactivity and low toxicity [6]. The recent literature reviewed the chemical structures and biological activities of 345 pregnane glycosides from 1984 to 2019 [7]. C21 steroids have multiple biological activities, such as anti-tumor, anti-epilepsy, anti-viral, anti-inflammatory, lowering cholesterol, protecting the liver and kidney, and scavenging free radicals. However, the mechanism by which these effects are achieved has not yet been elucidated [8].

Na^+^, K^+^-ATPase (NAK) is widely distributed in mammalian cell membranes and participates in ion exchange, and maintains the Na^+^-K^+^ balance and cellular osmotic pressure [9]. The α1 subtype (ATP1A1) is its catalytic subunit with Mg^2+^, ATP, Na^+^, K^+^, and ouabain binding sites [10]. Recent studies have found that any mutations in the NAK gene may cause greater physiological disturbances than the inactivation of ion pump functions. Clinically, the expression of ATP1A1 is higher in a large proportion of hepatocellular carcinoma (HCC) patients [11]. Downregulating the expression of ATP1A1 can significantly reduce the proliferation and migration of HCC cells and also promote cell apoptosis, reducing their tumorigenicity in vivo [12]. Therefore, ATP1A1 is a potential target for the treatment of HCC. Cardenolides, as NAK inhibitors, have been used in antitumor clinical research [13,14]. Even though it is a structurally similar compound, the notion as to whether C21 steroidal glycosides have an antitumor effect on NAK has not yet been reported [15]. Recently, we found that C21 steroids had an antiproliferation effect. Cynsaccatol L (**5**) [16,17], the most active compound among them, could affect the localization and degradation of ATP1A1. Therefore, we focused on the regulatory effect of **5** on ATP1A1 in HepG2 cells and on the specific antitumor mechanism.

Hepatocellular carcinoma (HCC) is a typical inflammatory tumor. In the tumor micro-environment (TEM), tumor-associated macrophages (TAMs) are an essential component of TEM, which plays an important role in the development of cancer progression [18]. Macrophages are transported by blood vessels to various tissues of the body and ultimately differentiate into different phenotypes in the local microenvironment [19] to exert immune stimulatory or immunosuppressive effects. M2 macrophages are generally the major phenotype of TAMs This is in addition to the anti-inflammatory effect of steroids, which has been widely reported. In the study, **5** could inhibit nitric oxide (NO) production in LPS-induced RAW264.7 cells. Therefore, we discussed the dual bioactivities of **5** on macrophages and HepG2 cells in co-cultivation.

In the present study, we found that **5** exerted anti-proliferation and pro-apoptotic effects in HepG2 cells. The apoptosis that was induced by **5** was associated with the inhibition of ATP1A1-AKT/ERK signaling pathways. In addition, **5** could up-regulate the cytokine levels and phagocytosis of macrophages, thereby altering the micro-environment of co-cultivation, which further promoted the apoptosis of HepG2 cells under co-culture conditions.

## 2. Results

### 2.1. Bioactivity of C21 Steroidal Glycosides from E.kansui

The cell viability of fractions A~E on HepG2 was detected (Table 1). The results showed that the anti-proliferation effect of fractions E and C was better than that of the other fractions. The half-inhibiting concentrations (IC_50_) of fractions C and E were 39.68 ± 4.53 μg/mL and 13.92 ± 3.28 μg/mL, respectively. Therefore, we sequentially tracked the chemical components of fractions E and C in turn and obtained 12 C21 steroidal glycosides, **1**–**6** and **7**–**12**, respectively. Compounds **1**–**12** exhibited different inhibitory activities on HepG2 cells (Table 2), wherein the IC_50_ values of compounds **1**, **2**, **5**, **6**, **7,** and **10** ranged from 12.55 ± 2.98 μM to 46.38 ± 3.09 μM. In addition, C21 steroidal glycosides also inhibited NO production in LPS-stimulated RAW264.7 cells (Table 3). It is worth noting that cynsaccatol L (**5**) showed the best inhibitory activity on HepG2 cells, as well as a significant inhibitory activity on NO production in LPS-stimulated RAW264.7 cells, which was 1000 times higher than that of the positive control drug (L-NMMA). According to the results, **5** was used for the following experiments.

### 2.2. Formatting of Mathematical Components

#### 2.2.1. Inhibition of the Proliferation and Migration of HepG2 Cells

**Figure 1:**
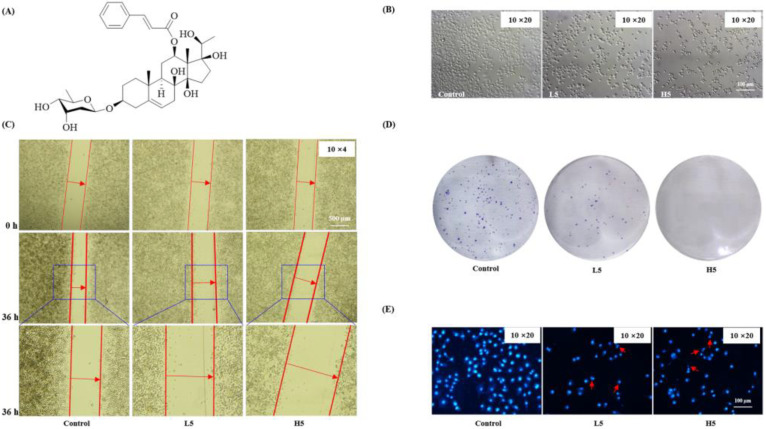
Compound **5** inhibited the migration and proliferation of HepG2 cells. L5, low concentration of **5** (12.5 μmol^−1^); H5, high concentration of 5 (25 μmol^−1^). (**A**) The structure of compound **5**. (**B**) The morphology of HepG2 cells was treated with **5** for 48 h. (**C**) The scratch test of **5** on HepG2 cells was detected for 36 h. (**D**) The colony test of HepG2 cells by **5** treatments was detected for 7 days. (**E**) Fluorescence images were detected by Hoechst staining. Red arrows pointed to the fragmented nucleus. The same concentrations of **5** were used in the following.

The structure of **5** is shown in Figure 1A (Figures S3 and S4). The effect of **5** on HepG2 was assessed by using a scratch test, colony formation assay, and fluorescence staining. Scratch and colony formation results showed that **5** greatly inhibited cell migration and proliferation (Figure 1B–D). Hoechst 33342 staining showed that **5** could significantly induce nuclear changes in HepG2 cells (Figure 1E). The above results demonstrated that **5** inhibited the proliferation and migration of HepG2 cells in vitro.

#### 2.2.2. Promotion of HepG2 Cell Apoptosis

**Figure 2:**
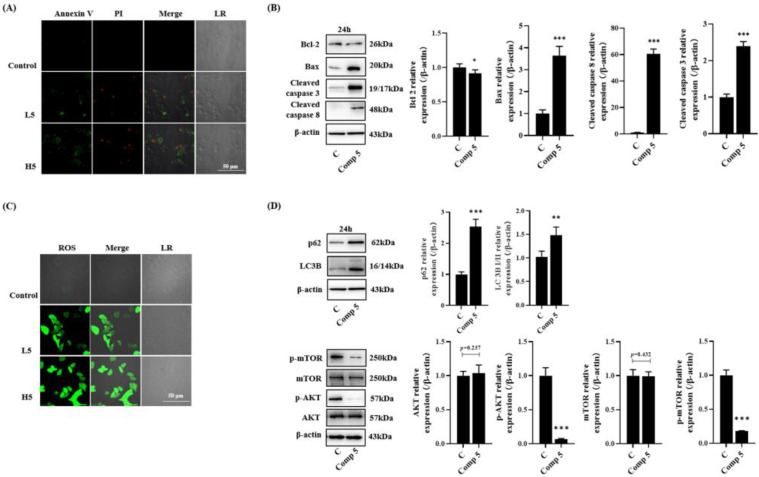
Compound **5** promoted the apoptosis of HepG2 cells. (**A**) Fluorescence image of cell apoptosis in HepG2 cells. The cells were double stained with Annexin V and PI. Early apoptotic cells were stained with Annexin V, and late apoptotic cells were stained with PI. (**B**) HepG2 cells were treated with Compound **5** (25 μmol∙L^−1^) for 24 h, and the protein expression was detected by western blot. (**C**) Fluorescence image of ROS in HepG2 cells. (**D**) HepG2 cells were treated with Compound **5** (25 μmol∙L^−1^) for 24 h, and autophagy-related protein expression and AKT/mTOR were detected by western blotting. Data are expressed as the mean ± standard deviation. * *p* < 0.05, ** *p* < 0.01, *** *p* < 0.001 vs. the control group.

The occurrence of apoptosis is characterized by morphological events and the activation of apoptotic signaling pathways. Herein, we detected the apoptosis events of HepG2 cells by fluorescence staining and Western blotting. Annexin V/PI staining showed that a value as low as 12.5 μmol∙L^−1^ **5** could significantly induce nuclear and cell membrane morphological changes in HepG2 cells (Figure 2A). Apoptosis-related proteins, such as Bax, cleaved caspase 3, and cleaved caspase 8, were increased, and Bcl-2 was decreased (Figure 2B). In addition, H2DCFDA staining also showed that **5** significantly increased ROS levels, which thus promoted apoptosis (Figure 2C). These findings indicated that **5** could accurately induce apoptosis.

It is known that autophagy and apoptosis can crosstalk and trigger proapoptotic signaling to promote cell death [20]. Therefore, we selected two autophagy-related proteins (LC3B and p62) and the AKT/mTOR signaling pathway in order to investigate whether autophagy was involved in cell death via a **5** treatment. As shown in Figure 2D, **5** inhibited the phosphorylation of AKT and mTOR, as well as upregulating the expression of LC3-B and p62. The inhibition of the AKT/mTOR signaling pathway and the high expression of LC3-B are the biomarkers of autophagy. In addition, p62 is involved in the ubiquitination of LC3 to block autophagy [21]. These results indicated that **5** could induce autophagy-related apoptosis in HepG2 cells.

### 2.3. Against HepG2 Cells the Inhibition of the ATP1A1-Related ERK and AKT Signaling Pathways

#### 2.3.1. Decreasing ATP1A1 Expression and Inhibiting NAK Activity

**Figure 3:**
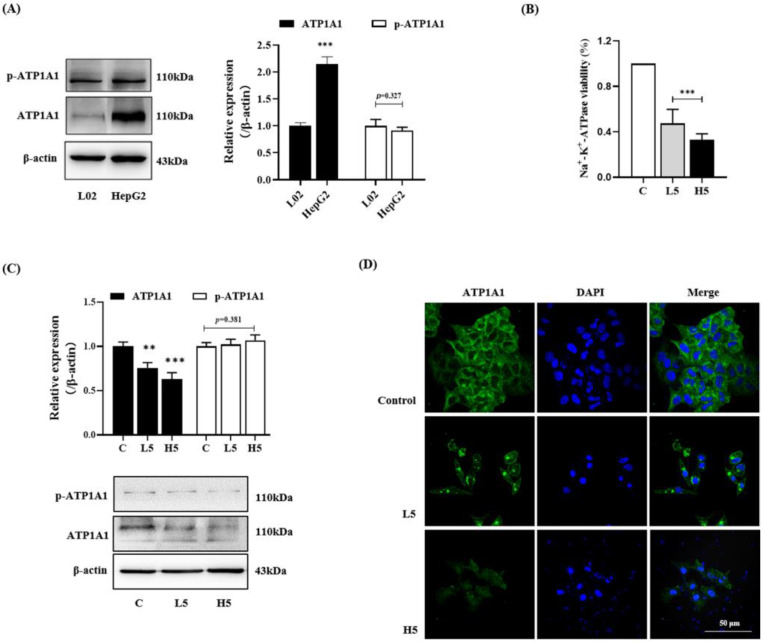
Compound **5** inhibited the activity of Na^+^-K^+^-ATPase and the level of ATP1A1 protein. (**A**) The expression and phosphorylation of ATP1A1 in L02 and HepG2 cells. (**B**) The effect of **5** on regulating the activity of Na^+^-K^+^-ATPase. (**C**) The effect of **5** on regulating the expression and phosphorylation of ATP1A1 in HepG2 cells. (**D**) Fluorescence image of ATP1A1 and DAPI in HepG2 cells. Data are expressed as the mean ± standard deviation. ** *p* < 0.01, *** *p* < 0.001 vs. the control group.

In a clinical setting, ATP1A1 is highly expressed in a large proportion of hepatocellular carcinoma (HCC) patients [22,23]. The down-regulation of ATP1A1 expression can inhibit the apoptosis and migration of hepatocellular carcinoma. Herein, as the structural analog of cardenolides, we further investigated the effect of **5** on the ATP1A1 target [24,25,26]. First, we verified the difference in ATP1A1 expression in L02 and HepG2 cells. When compared with the L02 cell group, ATP1A1 was over-expressed in HepG2 cells, but there was no significant difference in the phosphorylation of ATP1A1 (Figure 3A). In addition, unlike the effect of cardenolides, **5** not only inhibited the activity of NAK but also significantly reduced the level of ATP1A1 (Figure 3B,C). To further verify the impact of **5** on ATP1A1, the changes in the amount and location of ATP1A1 were detected by fluorescence staining. The results showed that, after a 5 treatment, the green fluorescence intensity of ATP1A1 on the cell membrane was significantly reduced in a dose-dependent manner (Figure 3D). In addition, ATP1A1 was evidently transferred into the cytoplasm from the membrane.

#### 2.3.2. Promotion of the Endocytosis and Degradation of ATP1A1 Protein

**Figure 4:**
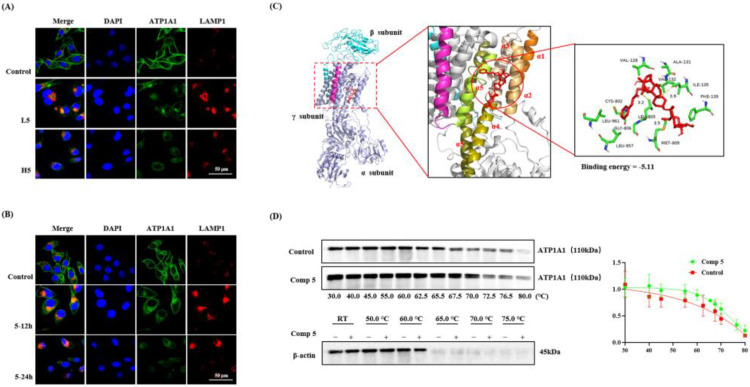
Compound **5** promoted the endocytosis and degradation of ATP1A1. (**A**) HepG2 cells were treated with 12.5 μmol∙L^−1^ or 25 μmol∙L^−1^ **5** for 48 h, and the lysosomal co-localization assay was performed with a confocal laser. (**B**) Cells exposed to 25 μmol∙L^−1^ **5** for 12 h or 24 h were detected by fluorescence staining. (**C**) **5** is depicted in red, and the chains of Na^+^, K^+^-ATPase α/β/γ are represented by light blue, cyan-blue, and magenta, respectively. The crystallography, atomic coordinates, and structure factors have been deposited in the Protein Data Bank, www.pdb.org (accessed on 1 February 2023) (PDB ID code 3KDP). (**D**) The CETSA binding assay of ATP1A1 and β-actin in the presence or absence of **5** (50 μmoL^−1^) at different temperatures was detected by western blot. The temperature-dependent melting curves and the apparent aggregation temperature were calculated by nonlinear regression. Values represent the mean ± SEM (N = 3 replicates).

Protein degradation is an important mechanism by which cells maintain protein homeostasis and signal transmission. The endolysosomal pathway is mainly responsible for the degradation of extracellular proteins and transmembrane proteins and regulates the signal transmission and antigen presentation. Therefore, to further trace ATP1A1 changes in HepG2 cells treated with **5**, we detected the effect of **5** on the degradation of ATP1A1 by a lysosomal colocalization assay. We observed a decrease in the fluorescence intensity of ATP1A1 on the cell membrane. Furthermore, **5** induced the ATP1A1 protein to cluster in the cytoplasm, which was consistent with the lysosomal location. These results showed that **5** could induce the endocytosis of the ATP1A1 protein, as well as the proteins that were degraded in a dose-dependent (Figure 4A) and time-dependent (Figure 4B) manner in lysosomes. In addition, to test whether **5** could bind to the active pocket of NAK, we predicted their binding ability by the use of the AutoDock4 software. As previously reported, the transmembrane helices αM1-6 of the α-subunit are the active pocket of Na^+^, K^+^-ATPase phosphoenzyme (E2P) that is exposed extracellularly, thus forming the high-affinity of E2P. We found that **5** mainly communicated with residues of αM1-4, such as VAL 128, VAL 132, ALA 131, ILE 125, PHE 139 (α1-2), CYS 802, LEU 805 MET 809, LEU 961, and GLY 806. The main forces of their interactions were determined to be the conjugation effect and hydrogen bonding force (Figure 4C). Compared with the control group, the CETSA results showed that the ATP1A1 protein was degraded with the temperature increasing, but the denaturation temperature of the compound group was higher than that of the control group. Moreover, **5** did not change the denaturation temperature of β-actin (Figure 4D). These findings showed that **5** when combined with ATP1A1 and induced the endocytosis and degradation of the ATP1A1 protein.

#### 2.3.3. Inhibition of ATP1A1-Related AKT and ERK Signaling Pathways

**Figure 5:**
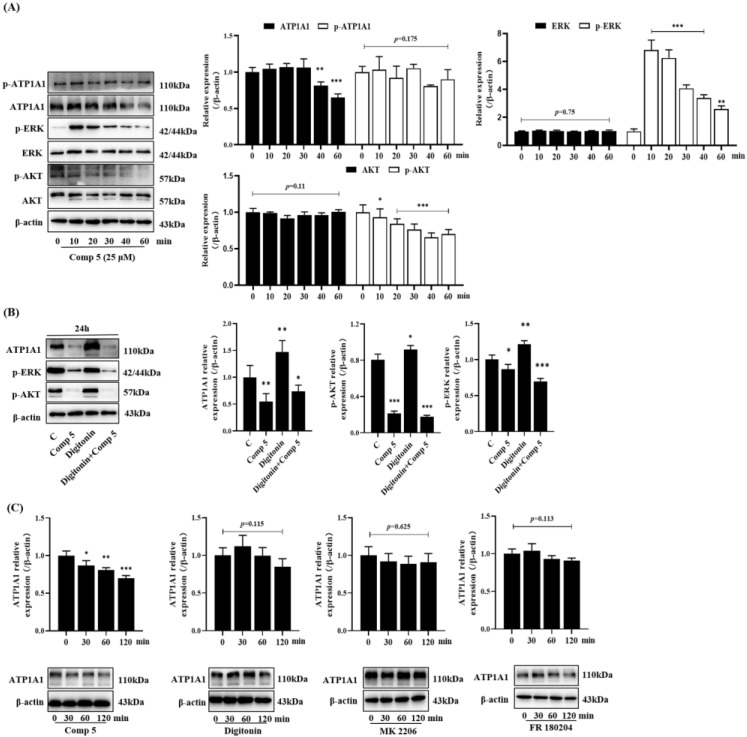
Compound **5** downregulated ATP1A1-related AKT and ERK signaling pathways. (**A**) HepG2 cells were treated with 25 μmol∙L^−1^ **5** for the indicated times, and the expression of AKT, ERK, ATP1A1, and their phosphorylation were measured by western blotting. (**B**) Cells were exposed to 25 μmol∙L^−1^ **5** for 24 h in the absence or presence of 10 nmol∙L^−1^ digitonin, phosphor-AKT, phosphor-EKR, and ATP1A1 were detected by western blot. (**C**) HepG2 cells were treated with or without **5** (25 μmol∙L^−1^), digitonin (10 nmol∙L^−1^), MK 2206 (40 nmol∙L^−1^), or FR 180204 (200 nmol∙L^−1^) for the indicated times, and the expression of ATP1A1 was detected by western blot. Data are expressed as the mean ± standard deviation. * *p* < 0.05, ** *p* < 0.01, *** *p* < 0.001 vs. control group.

ATP1A1 has involved in tyrosine kinase-dependent cellular signal transduction. As downstream signaling molecules, AKT and ERK play an important role in a series of defined proliferation, differentiation, and apoptosis events [27,28]. Based on research, ATP1A1 was reported to modulate the ERK and AKT signaling pathways. Therefore, we detected the expression and phosphorylation of AKT and ERK by Western blotting in order to verify whether ERK and AKT inactivation was consistent with ATP1A1 downregulation by a **5** treatment. First, HepG2 cells were treated with **5** for 0~60 min to assay the changes in proteins. The results showed that the phosphorylation of AKT and ERK and the expression of ATP1A1 were all decreased by a **5** treatment (Figure 5A). Then, a NAK inhibitor (digitonin) was used to observe whether the inactivation of AKT and ERK was due to the down-regulation that occurs following the phosphorylation of ATP1A1. As shown in Figure 5A,B, the level of ATP1A1 and the phosphorylation of AKT and ERK were decreased after 10 min of treatment in the **5** group and the co-treatment group, but not in the digitonin group. The presence of digitonin did not suppress the effect of **5**. Na^+^/K^+^-ATPase inhibitor (digitonin), AKT inhibitor (MK 2206), and ERK inhibitor (FR 180204) were used to investigate whether the downregulation of ATP1A1 expression was induced by AKT and ERK inactivation. As shown in Figure 5C, the expression of ATP1A1 was decreased after a **5** treatment, but this was not similarly observed in the other groups. In summary, this suggested that the **5**-induced inactivation of AKT and ERK was due to the down-regulation of ATP1A1 expression.

### 2.4. Further Promotion of the Apoptosis of HepG2 Cells by Regulating Macrophage Function

#### 2.4.1. Promotion of HepG2 Cell Apoptosis

**Figure 6:**
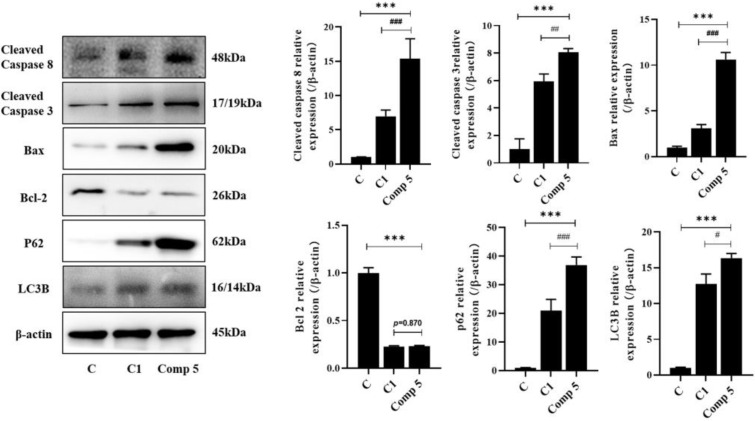
The effect of Compound **5** on HepG2 cells or HepG2 and RAW264.7 co-cultivation. C: HepG2 and RAW264.5 co-cultivations. C1: HepG2 cells were treated with 25 μmol∙L^−1^ of **5**. Comp **5**: HepG2 and RAW264.5 co-cultivations were treated with 25 μmol∙L^−1^ of **5**. Cells were treated by **5** for 48 h, and the expression of cleaved-caspase 3 and 8, LC3-B, Bax, Bcl-2, and p62 was detected by western blot. Data are expressed as the mean ± standard deviation. *** *p* < 0.001 vs. C group. ^#^ *p* < 0.05, ^##^ *p* < 0.01, ^###^ *p* < 0.001 vs. C1 group.

Compound **5** showed the dual effects of direct anti-proliferation effects and regulation of the macrophage response (Table 1 and Table 3). Therefore, whether **5** had dual effects in terms of being able to exert the anti-HepG2 effect is to be the content of our further study. Herein, we observed the effect of **5** on the apoptosis of HepG2 cells in co-cultivation. The co-culture of RAW264.7 and HepG2 cells is a common experimental method to analyze the interaction between two kinds of cells [29,30]. Compared with the co-cultivation group (C group) and the HepG2 cell group (C1 group), **5** could significantly upregulate the expression of cleaved-caspase 3, cleaved-caspase 8, LC3B, p62, and Bax, as well as down-regulating Bcl2 expression (Figure 6). The results indicated that **5** could further promote the apoptosis of HepG2 cells that are cocultured with macrophages.

#### 2.4.2. Regulated Macrophage Function in Cocultivation

**Figure 7:**
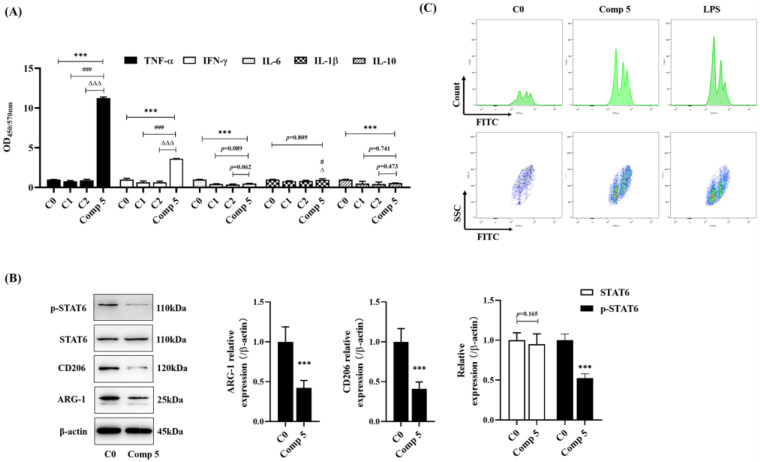
The effects of Compound **5** on HepG2 cells or HepG2 and RAW264.7 co-cultivation. C0: HepG2 and RAW264.5 co-cultivations. C1; RAW264.7 cells; C2; RAW264.7 cells were treated with 25 μmol∙L-1 of 5; Comp **5**: HepG2 and RAW264.5 co-cultivations were treated with 25 μmol∙L^−1^ of **5**. Cells were treated for 48 h. (**A**) The levels of TNF-α, IFN-γ, IL-6, IL-1β and IL-10 by ELISA kits. (**B**) The expression of ARG-1, COX2, STAT6, and p-STAT6 were detected by Western blot. (**C**) C: HepG2 cells and RAW276.7 cells were co-cultivated and were treated with **5** (Comp **5**) or not. RAW264.7 cells were treated with 1 μg/mL LPS only (LPS). The cells were analyzed by Flow cytometry. Data are expressed as mean ± standard deviation. *** *p* < 0.001 vs. C0 group; ^#^ *p* < 0.05, ^###^ *p* < 0.001 vs. C1 group; ^∆^ *p* < 0.05, ^∆∆∆^ *p* < 0.001 vs. C2 group.

To determine the effect of **5** on HepG2 cells in co-cultivation, we further detected the cytokine levels and the phagocytic ability of RAW264.7 cells. Compared with C0, C1, and C2 groups, **5** could significantly upregulate the levels of TNF-α and IFN-γ (Figure 7A). Compared with the C0 group, the levels of IL-6 and IL-10 decreased significantly, but there was no obvious difference compared with the C1 and C2 groups. Further, **5** downregulated the expression of CD206 and ARG-1 in cells in the supernatant (Figure 7B), which meant that the high expression of TNF-α and IFN-γ promoted the apoptosis of HepG2 cells and that the reduction in IL-6 levels inhibited the IL-6/STAT signaling pathway to weaken the antiapoptotic effect of HepG2 cells. In addition, **5** significantly upregulated the phagocytic ability of macrophages (Figure 7C) when compared with that of the control group. These results indicated that **5** could regulate the secretion of inflammatory cytokines in co-cultivation and promote the phagocytosis of macrophages, thereby further promoting the apoptosis of HepG2 cells.

## 3. Discussion

As a traditional Chinese medicine, *E. kansui* was first recorded in “Shen Nong’s Materia Medica”. It reportedly had the functions of dispelling symptoms, dispelling knots, and benefiting water and valleys. *E. kansui* is widely used in the treatment of ascites, edema, and pleural fluid effusion [31]. Recently, its clinical applications have extended beyond these diseases, as it is also used in the treatment of leukemia, liver cirrhosis, tumors, and other diseases. To date, nearly 100 compounds have been isolated and identified from *E. kansui* [32], and these compounds exhibit a wide range of biological activities [33]. In the present study, we evaluated the anti-proliferative activity of different fractions of *E. kansui* and further isolated and identified the components in fractions E and C, which showed a more pronounced inhibitory effect. By bioactivity tracking, we found that steroids also played an important role in the anti-HepG2 activity. Twelve C21 steroidal glycosides **1**–**6** and **7**–**12** were obtained from fractions E and C, respectively. Among them, **1**, **2**, **5**, **6**, **7,** and **10** had anti-proliferative activity on HepG2 cells, especially **5**. In addition, these steroidal glycosides also showed significant anti-inflammatory activity; among them, the inhibitory activity of **5** on NO production in LPS-stimulated RAW264.7 cells was 1000 times higher than that of the positive control. Therefore, we detected the bioactivity of **5** on HepG2 cells and then conducted co-cultivation to study the possible anti-HepG2 mechanisms of **5**.

Researchers report that C21 steroidal glycosides can inhibit tumor cells by regulating the Wnt/β-catenin signaling pathway, TLR4/MyD88/NF-κB, AKT signaling pathway, PI3K/AKT/mTOR signaling pathway, and Hippo pathway [8,34,35]. In the present study, we found that **5** could inhibit the migration and proliferation of HepG2 cells, promote morphological changes, promote the caspase cascade, induce mitochondrial dysfunction and ROS accumulation, and upregulate the expression of associated proteins, such as Bax and cleaved caspase 3/8. To elucidate its anti-HepG2 molecular mechanism, we focused on the effect of C21 steroidal glycosides on ATP1A1 (Figure S1), which is a novel antitumor target of HCC. Clinically, ATP1A1 is highly expressed in a large proportion of HCC, and its level is closely related to clinical stage and prognosis [36]. It must be noted that silencing or inhibiting the expression of ATP1A1 can inhibit the proliferation and migration ability of cancer cells. First, we verified the expression of ATP1A1 in HepG2 cells and L02 cells. Then, we observed that **5** not only inhibited the pump activity of NAK but also promoted the endocytosis and degradation of the ATP1A1 protein. The cytoplasmic transport and degradation of ATP1A1 could regulate the downstream AKT and ERK signaling pathways, thereby inhibiting the proliferation and differentiation of HepG2 cells. We suggest that **5** induced the downregulation of ATP1A1, thereby inhibiting the AKT and ERK signaling pathways and exerting its anti-HepG2 effect. The effect of **5** on anti-proliferation and downregulation of ATP1A1 expression in another hepatoma cell line (Huh-7) was also preliminarily verified by experiments (Table S1, Figure S2).

**Figure 8:**
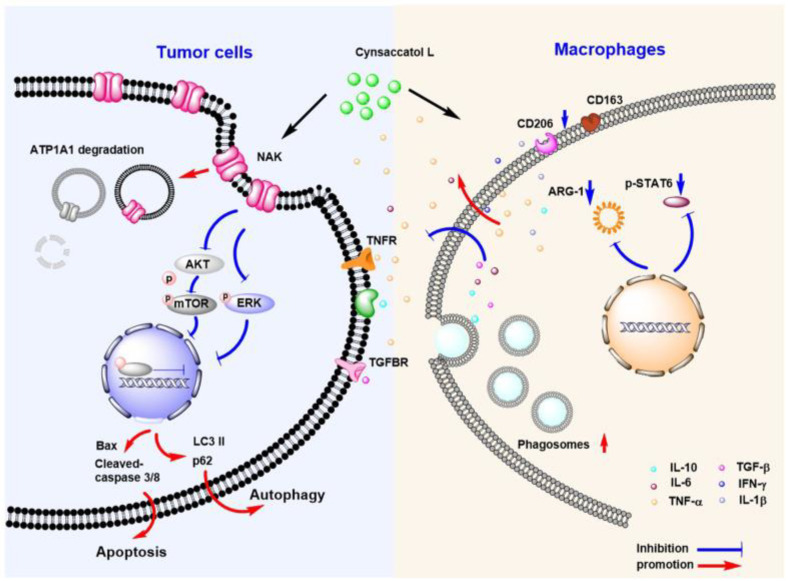
Schematic diagram of the dual anti-proliferation activity of cynsaccatol L.

In addition, **5** showed dual effects, including direct anti-proliferative effects and the regulation of the macrophage inflammatory response. Whether **5** assisted in the anti-HepG2 effect by modulating macrophages was the focus of our attention. In the present study, we used a cell coculture system to further study the anti-HepG2 effect of **5**. The results showed that, when conducting a co-cultivation, **5** further enhanced HepG2 apoptosis and promoted the expression of apoptotic proteins, such as Bax and cleaved caspase 3 and 8, in cocultivation. The effect was more significant than that of **5** alone. When tracking changes in macrophages, we found that **5** could elevate the levels of inflammatory cytokines, such as TNF-α and IFN-γ, and decrease the release of IL-6 and IL-10. TNF-α is an important regulator of inflammation, and macrophages are one of the main cells that secrete them. High expression of TNF-α can induce apoptosis. IL-6 is also one of the main cytokines that bind to the IL-6 receptor to activate the STAT signaling pathway and can induce macrophages to differentiate into M2 type [37], thereby promoting the proliferation of tumor cells and resisting apoptosis. Therefore, the regulatory effect of **5** on the expression of TNF-α and IFN-γ promoted the apoptosis of HepG2 cells and inhibited the IL-6/STAT signaling pathway, weakening the anti-apoptotic effect of HepG2, thereby promoting the apoptosis of HepG2 cells. In addition, **5** decreased the biomarkers of M2 macrophages, such as CD206 and ARG-1. Here we mainly discussed the influence on cytokine secretion and the phenotypic change of macrophages under co-culture conditions. the production of nitric oxide was not detected. However, nitric oxide plays complex roles in the occurrence and development of tumors [38,39]. This is a topic worthy of further investigation and we hope to investigate it further in future studies. Meanwhile, **5** also increased the phagocytic ability of RAW264.7 cells. These changes in macrophages resulted in significant changes in co-cultivation, which might further trigger HepG2 apoptosis. The possible mechanisms are shown in Figure 8. In conclusion, our results suggested that **5** might have the antitumor mechanism of directly inducing tumor cell apoptosis by inhibiting ATP1A1-related AKT-ERK signaling pathways and regulating the function of macrophages in co-cultivation.

## 4. Materials and Methods

### 4.1. Plant Material

*Euphorbia kansui* T. N. Liou ex T. P. Wang was collected in Linfen, Shanxi, in May 2019 and was identified by Professor Guo Shiming, Yunnan Academy of Traditional Chinese Medicine. The specimen was preserved in the Resource Medicinal Chemistry Laboratory of the School of Life Science and Technology, Kunming University of Science and Technology; and the specimen number is GS201905.

### 4.2. Extraction and Isolation

The powdered roots of *E. kansui* (25 kg) were extracted three times with 95% EtOH (3 × 100 L, 48 h, each) at room temperature. Then, the filtrate was concentrated to give an extract, which was partitioned with EtOAc and water. The EtOAc extract (448 g) was subjected to silica gel (20~300 mesh) CC, and then successively eluted with petroleum ether (PE)/EtOAc (45:1→0:1) and CHCl_3_/MeOH (1:1) to give five fractions (A~E). According to the bioactivities of fractions A~E, fractions E and C were further isolated. Fraction C was separated by RP-18 (50%→100% MeOH/H_2_O) to give ten fractions (C1~C10). Compounds **7** (2.9 mg) and **8** (8.2 mg) were acquired from subfractions C-2-1 (2.01 g) and C-2-2 (1.37 g), respectively. Then, semipreparative HPLC with CH_2_Cl_2_: IPA 200:1 and CHCl_3_/MeOH 150:1 yielded compound **10** (10.4 mg). Compound **11** (3.3 mg) was separated from C4-5 (0.9 g) by silica gel (PE/acetone 12:1). C-4-6 (1.15 g) and was subjected to silica gel CC (PE/isopropanol 50:1) to yield compound **12** (3.3 mg). Compound **9** (10.0 mg) was purified from subfraction C4-3-2 by silica gel (CH_2_Cl_2_: Isopropanol 200:1).

Fraction E was subjected to medium-pressure RP-18 (MeOH/H_2_O, 40%→100%) to give eight fractions (E1~E8). E2 (1.2 g) was subjected to Sephadex LH-20 gel (CHCl_3_/MeOH 1:1) and silica gel (CHCl_3_/MeOH 50:1) to yield compounds **2** (3.1 mg) and **3** (32.5 mg). Compound **1** (6.1 mg) was isolated from E-3 (60 mg) by silica gel (CHCl_3_/isopropanol 70:1). E-6-2 (1.53 g) was subjected to silica gel (CHCl_3_/MeOH 60:1) and then purified by semipreparative HPLC (55% MeOH/H_2_O) to obtain compound **4** (10.6 mg). Compound **5** (3.8 mg) was obtained from subfraction E7-1-1 by semipreparative HPLC (55% MeOH/H_2_O). E-6-3-2-2 (15.0 mg) was subjected to silica gel CC (CHCl_3_/MeOH 100:1) and then purified by semipreparative HPLC (57% MeOH/H_2_O) to yield compound **6** (10.0 mg).

### 4.3. Reagents

C21 steroidal glycosides were extracted and purified from *E.kansui* and supplied at 95% purity by the Key Laboratory of Resources and Medical Chemistry, Kunming University of Science and Technology. The structures of compounds **1**–**12** were identified by Dr. Jian-chun Li (Kunming University of Science and Technology). The purity (>98%) of those C21 steroidal glycosides was determined by HPLC and NMR tests. Compounds **1**–**12** and adriamycin (Solarbio, Beijing, China) were subsequently made up to a stock of 50 mmol∙L-1 in dimethyl sulfoxide (DMSO, Aladdin, Shanghai, China) and then stored at 4 °C. ATP1A1, p-ATP1A1, ERK, p-ERK, AKT, p-AKT, p62, LC-3 (I/II), Bcl-2 and Bax antibodies were purchased from CST (Cell Signaling Technology, Danvers, MA, USA); cleaved caspase 8 was purchased from Affinity (NanJing, China); β-actin from Servicebio (Wuhan, China); the FITC rabbit antibody from ZhongshanJinqiao Biotechnology (Beijing, China); DAPI from Beyotime (Beijing, China); the Annexin V/PI kits from BD (Franklin Lakes, NJ, USA); and the Hoechst from Sigma Aldrich (St. Louis, MO, USA).

### 4.4. Cell Culture

L02, HepG2, and RAW264.7 cells were incubated in DMEM (Gibco, Carlsabad, CA, USA). Then, they were supplemented with 10% FBS (Gibco, Carlsbad, CA, USA) and 100 IU/mL streptomycin-penicillin (Solarbio, Beijing, China) in a CO_2_ chamber at 37 °C and 95% humidity. The morphology of the cells was observed using a reversed microscope (Olympus Corporation, Shinjuku, Japan) at × 200 magnifications. The Tissue Culture Plate Insert (LABSELECT, Beijing, China, Lot: 20421087A) chambers were used for HepG2 and RAW264.7-cell co-cultivation.

### 4.5. MTT Assay 

HepG2 cells (1 × 10^4^ cells/well) or RAW264.7 cells (1 × 10^8^ cells/well) were incubated in 96T wells for 24 h. Then, HepG2 cells were treated with compounds or Adriamycin for 48 h. In addition, RAW264.7 cells were treated with compounds for 24 h. Then, 0.5% MTT (Sigma, Saint Louis, MO, USA) was added to 20 μL/well for 3.5 h. The supernatant was discarded, and DMSO (150 μL/well) was added to dissolve the purple formazan. Cells were agitated for 15 min at room temperature, and the absorbance at 490 nm was recorded by SpetraMax M2 (Molecular Devices, San Jose, CA, USA).

### 4.6. Cell Migration and Colony Formation Assay

HepG2 cells (8 × 10^4^ cells/well) were incubated in 24-well plates for 24 h. Cells were treated with a compound for 36 h after making a uniform straight from scratch with a 200 μL pipette tip. Cell motility was detected by measuring the wound closure distance after 36 h. Images were recorded using an inverted microscope (Olympus CK40, Shinjuku, Japan). HepG2 cells (600 cells/well) were incubated in 6-well plates and treated with the compound. One week later, visible colonies had formed. Then, the colonies were fixed with 4% polymethanol paraformaldehyde and stained with crystal violet (0.005%). The number of colonies was calculated by using a microscope.

### 4.7. Protein Kinase Inhibitor 

An Na^+^/K^+^-ATPase inhibitor (digitonin), AKT inhibitor (MK 2206), and ERK inhibitor (FR 180204) were purchased from Biotech (Beijing, China). These agents were stored at −20 °C, and the cells were preincubated with the inhibitor for 4 h prior to treatment with the compounds. Digitonin, MK 2206, and FR 180204 were used at concentrations of 10, 40, and 200 nmol/L, respectively.

### 4.8. Fluorescence Staining

HepG2 cells (4 × 10^5^ cells/well) were incubated in confocal dishes with DMEM for 24 h and then treated with compounds for 48 h. Then, the cells were treated with 0.1% Triton X-100 for 10 min and washed with PBS five times. Finally, the cells were dyed with anti-ATP1A1 at 4 °C overnight. Nuclear staining with DAPI (1 μg∙mL^−1^) was added for 7 min in the dark, and the cells were washed. The stained images were captured under a laser scanning confocal microscope (Nikon A1 AIR MP+, Tokyo, Japan). HepG2 cells (5 × 10^5^ cells/well) were incubated in 6-well plates in complete DMEM. Cells were treated with compounds for 24 h. Then, the cells were stained with Annexin V/PI for 30 min at 4 °C in the dark, and apoptosis analysis was carried out by laser scanning confocal microscopy.

### 4.9. Na^+^-K^+^-ATPase Analysis

HepG2 cells were collected in centrifuge tubes, and every 5 million cells were added to 1 mL reagent to sonicate the cells (power 20%, ultrasound 3S, interval 10 s, repeat 30 times). Then, the samples were centrifuged at 8000 rpm/min for 10 min at 4 °C. The supernatant was then collected and placed on ice for testing. The operation was performed according to the instructions of the Na^+^-K^+^-ATPase enzyme activity detection kit (Solarbio, Beijing, China).

### 4.10. Western Blot Analysis

Protein samples were prepared using a RIPA lysis buffer. The concentration of the protein was analyzed using a nucleic acid and protein microanalyzer (Molecular Devices, San Jose, CA, USA). SDS–PAGE was performed to separate the proteins, which were then electrotransferred onto a PVDF membrane. The membrane was incubated with antibodies at 4 °C overnight. Next, the membrane was washed and incubated with an HRP-conjugated secondary antibody (Beyotime, BeiJing, China). Finally, signals were detected by the ECL method (Beyotime, BeiJing, China) and analyzed using ImageJ software.

### 4.11. Flow Cytometry

RAW264.7 cells (1.5 × 10^5^ cells/well) and HepG2 cells (4 × 10^5^ cells/well) were incubated in a Tissue Culture Plate Insert (LABSELECT, Hangzhou, China) at 37 °C and 95% humidity. Raw264.7 cells were incubated in 12-well plates, and HepG2 cells were incubated in Transwell chambers. After 24 h, the cells were treated with the compound for 48 h. Then, the chamber was discarded, and RAW264.7 cells were washed twice with PBS and a fresh medium. Next, 1.5 mL/well and 100 μL/well FluoSpheresTM carboxylate (Invitrogen, Carlsbad, CA, USA) were added to the medium for 2 h in the dark. Cells were washed twice with PBS, harvested, and then resuspended in 0.5 mL PBS. Finally, the cells were measured by a FACSCalibur (Becton Dickinson, Franklin Lakes, NJ, USA). Data were analyzed using FlowJo software.

### 4.12. Measurement of Nitric Oxide and Cytokines

RAW264.7 cells (8 × 10^4^ cells/well) were incubated in 96T wells with DMEM for 24 h and then treated with compounds and LPS (1 μg/mL) for 24 h; this included the control group and LPS group. Then, the cell supernatant was collected for nitric oxide production assay. Next, 100 uL/well PBS was added to 96T wells, then 0.5% MTT (Sigma, Saint Louis, MO, USA) was added to 20 μL/well for 3.5 h. DMSO (150 μL/well) was added to dissolve the purple formazan. Cells were agitated for 15 min at room temperature, and the absorbance at 490 nm was recorded by SpetraMax M2 (Molecular Devices, San Jose, CA, USA). The NO inhibition rate (%) = (ODLPS group − ODcompound group)/(ODLPS group-ODcontrol group) × 100%. The IC50 value refers to the inhibitory of nitric oxide production of LPS-stimulated RAW264.7 cells. Then, 70 uL of each cell supernatant sample was added into a new 96T plate, and Griess A and Griess B were added separately. Then, the absorbance value at 540 nm was detected and then brought into the standard curve to assay the concentration of NO. The cell survival rate (%) = (ODcompound group − ODblank group)/(ODcontrol group − ODblank group) × 100%. The half concentration of the cell death (CC50) value refers to the concentration of compound **5** when 50% of RAW264.7 cells die. The iNOS inhibitor (NG-monnomycin-l-arginine, l-NMMA) was selected as the positive control. The levels of cytokines (IL-1β, IFN-γ, IL-10, TNF-α, and IL-6) in the cell supernatant were determined using an ELISA kit (Novus Biologicals, Shanghai, China, Lot: 834472, 770118, 772051, 486956, 602865). When the process was conducted according to the manufacturer’s instructions, the absorbance was read at 450 nm/570 nm in a SpectraMax 340PC microplate spectrophotometer (Molecular Devices, San Jose, CA, USA).

### 4.13. Statistical Analysis 

Data are presented as the mean with standard deviation (SD). Independent two-sample t-tests were used to compare the differences between the two groups. Further, one-way ANOVA with the least significant difference (LSD) test for post-host comparisons was used to compare the differences between three or four groups. A P value below 0.05 indicated statistical significance. The statistics were analyzed with SPSS 21.0 software (SPSS Inc., Chicago, IL, USA).

## Figures and Tables

**Table 1:** Cell viabilities of HepG2 cells treated by fractions A–E.

Drug	HepG2 IC_50_ (μg/mL)
Control	-
Adr ^1^	5.98 ± 2.06
A	>100
B	>100
C	39.68 ± 4.53
D	63.72 ± 6.01
E	13.92 ± 3.28

^1^ Adr: adriamycin.

**Table 2:** Cell viability of HepG2 cells treated by compounds **1**–**12**.

Compounds	Name	HepG2 IC_50_ (μM)
Control	-	-
Adr ^1^	-	6.93 ± 1.23
**1**	Eupokanu G	46.38 ± 3.09
**2**	Cynotophylloside B	41.11 ± 6.05
**3**	Deacylmetaplexigenin	>50
**4**	Kidjolanin	>50
**5**	Cynsaccatol L	12.61 ± 2.13
**6**	Kidjoranin-3-O-β-D-cymaropyranoside	27.30 ± 3.48
**7**	Wilfoside G	12.55 ± 2.98
**8**	Cynotophylloside J	>50
**9**	Maslinic acid	>50
**10**	Kidjoranin 3-O-α-diginopyranosyl-(1→4)-β-cymaropyranoside	22.73 ± 4.02
**11**	Kidjoranin 3-O-β-digitoxopyranoside	>50
**12**	Caudatin 3-O-β-D-digitoxopyranoside	>50

^1^ Adr: adriamycin.

**Table 3:** Cell viability of RAW264.7 cells treated with compounds 1–12 and the NO inhibition activities on LPS-stimulated RAW264.7 ($\bar{x}$ ± s, *n* = 3).

Compounds	RAW264.7		Compounds	RAW264.7	
	CC_50_^1^ (μM)	IC_50_ ^2^ (μM)		CC_50_ ^1^ (μM)	IC_50_ ^2^ (μM)
Control	-	-	**7**	26.15 ± 2.22	1.77 ± 0.34
LPS ^3^	-	-	**8**	>50	17.39 ± 3.01
**1**	>50	>50	**9**	>50	17.38 ± 2.21
**2**	>50	9.10 ± 2.27	**10**	24.12 ± 3.24	2.78 ± 0.44
**3**	>50	>50	**11**	>50	>50
**4**	>50	30.72 ± 4.08	**12**	>50	>50
**5**	30.12 ± 3.14	0.02 ± 0.02	l-NMMA ^4^	>50	21.22 ± 3.26
**6**	35.24 ± 5.12	3.32 ± 0.43	-	-	-

^1^ Cell survival rate (%) = (OD_drug_ − OD_blank_)/(OD_control_ − OD_blank_) × 100%. CC_50_ value refers to the half inhibitory of RAW264.7 cells. ^2^ NO inhibition rate (%) = (OD_LPS_ − OD_drug_)/(OD_LP_S − OD_control_) × 100%. The IC_50_ value refers to the inhibitory nitric oxide production of LPS-stimulated RAW264.7 cells. ^3^ LPS: Lipopolysaccharide. ^4^ l-NMMA: NG-monnomycin-l-arginine, iNOS inhibitor. Positive control substance in RAW264.7.

## Data Availability

Not applicable.

## Supplementary Materials

The following supporting information can be downloaded at: https://www.mdpi.com/article/10.3390/molecules28062830/s1, Figure S1: Molecular docking results of compounds **7** and **10** with Na^+^, K^+^-ATPase α subunits title; Figure S2: Compound **5** inhibited the proliferation and the level of ATP1A1 protein in Huh-7 cells; Figure S3: The 1H NMR spectrum of compound **5**; Figure S4: The ^13^C NMR spectrum of compound **5**; Table S1: Cell viability of Huh-7 cell treated by compound **5**.

## Sample Availability

Samples of the compounds are available from the authors.
