# Supplementary material for: Bioactive C21 Steroidal Glycosides from Euphorbia kansui Promoted HepG2 Cell Apoptosis via the Degradation of ATP1A1 and Inhibited Macrophage Polarization under Co-Cultivation
Source: Molecules. 2023 Mar 21;28(6):2830. doi: 10.3390/molecules28062830 (PMC10058894; doi:10.3390/molecules28062830)
Supplement: Supplementary file 1 [file molecules-28-02830-s001.zip › molecules-2244329-supplementary.pdf]

molecules

Supplementary Material

1. The binding ability to NAK of compound 7 and 10

As the structural analogues of compound **5**, we tested the binding ability of compounds **7** and **10** to the active pocket of NAK. Their binding abilities were analysis by AutoDock4 software. The result showed that the binding energy of **7** (-4.50 kcal·mol^{-1}) and **10** (-3.00 kcal·mol^{-1}) were higher than **5** (-5.11 kcal·mol^{-1}), and the binding position of **7** was closely to that of **5**. The mainly communicated with residues of αM1-4, such as VAL 128, VAL 132, ALA 131, ILE 125, PHE 139, CYS 802, LEU 805, MET 809, LEU 961 and GLY 806. But the binding position of **10** was different with **5** and **7**. The mainly communicated with residues of αM2-5, such as ILE 135, PHE 139, TYR-142, CYS 802, LEU 805, MET 809, LEU 961 and GLY 806. The main forces of their interactions were the conjugation effect and hydrogen bonding force (Figure S1). Based on this, we predicated compounds **7** and **10** might exert the similar mechanistic effects of **5**.

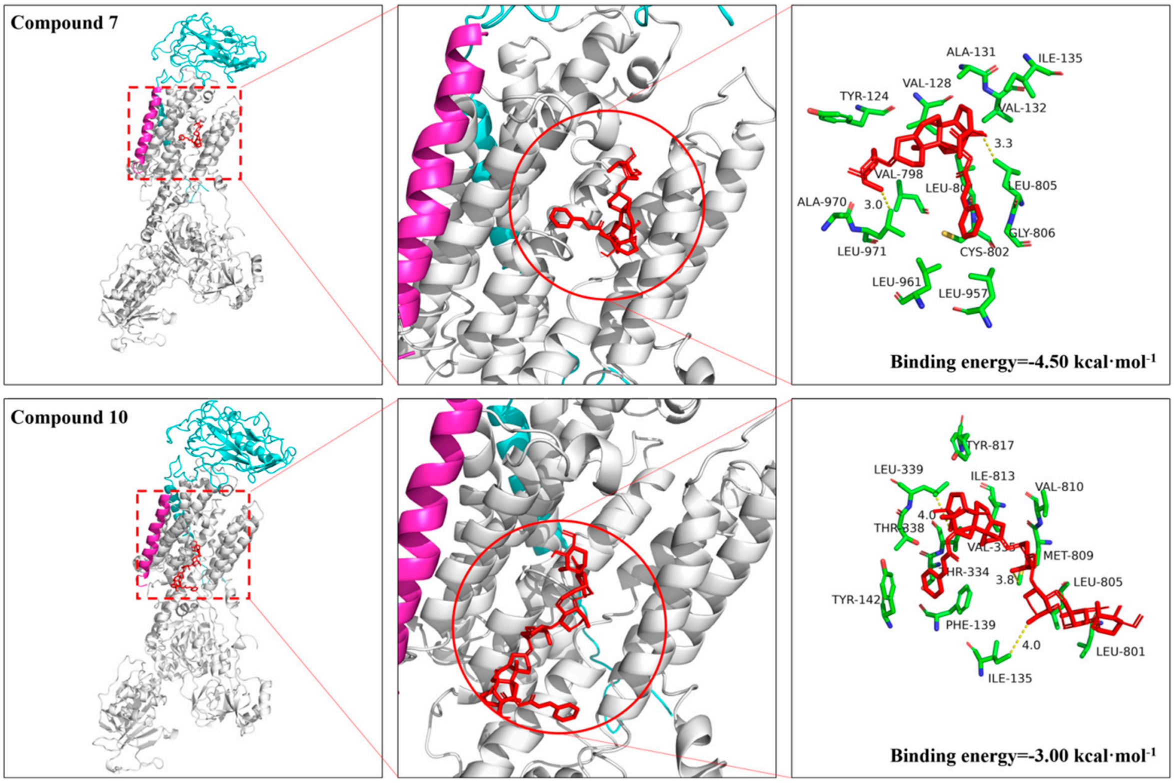

Figure S1 Molecular docking results of compounds 7 and 10 with Na^+, K^+-ATPase α subunits

Compounds **7** or **10** are depicted in red, and the chains of Na^+, K^+-ATPase α/β/γ are represented by light blue, cyan-blue and magenta, respectively. The crystallography, atomic coordinates, and structure factors have been deposited in the Protein Data Bank, www.pdb.org (PDB ID code 3KDP).

2. The anti-proliferation of compound 5 on Huh-7 cells

We tested another hepatoma cell line (Huh-7) to validate effect of compound **5**. L02 (normal hepatocytes), HepG2 and Hub-7 cells were used to discuss the effect of **5**.

First, the cytotoxicity of **5** on Huh-7 cells was detected by MTT assay, and the result showed that IC_{50} of **5** on Huh-7 cell was (24.49±233) μM (Table S1). The morphology and apoptosis of the cells by reversed microscope and flow cytometry, respectively. The results showed that **5** could significantly induce apoptosis of Huh-7 cells (Figure S2A and B).

Second, the expression and phosphorylation of ATP1A1 were detected by Western blot analysis in L02, HepG2 and Huh-7 cells. Compared with L02 group, the expression of ATP1A1 was higher in HepG2 and Huh-7 cells, but there was no significant difference in the phosphorylation of ATP1A1 (Figure S2C). It indicated that ATP1A1 was over-expressed in Huh-7 cells, which was consistent with HepG2 cells.

At last, the effect of **5** on the expression and the phosphorylation of ATP1A1 in Huh-7 cells was detected. The result showed that **5** significantly reduced the level of ATP1A1 in Huh-7, but not affect the phosphorylation of ATP1A1. The result was consistent with that observed in HepG2 cells (Figure S2D). Therefore, we suggested that ATP1A1 might be the target of **5** anti-hepatoma cell proliferation.

Table S1 Cell viability of Huh-7 cell treated by compound 5

**Drug**	**Huh-7**
	**IC_{50} (μM)**
Control	-
Adriamycin	4.49±0.96
**5**	24.49±2.33

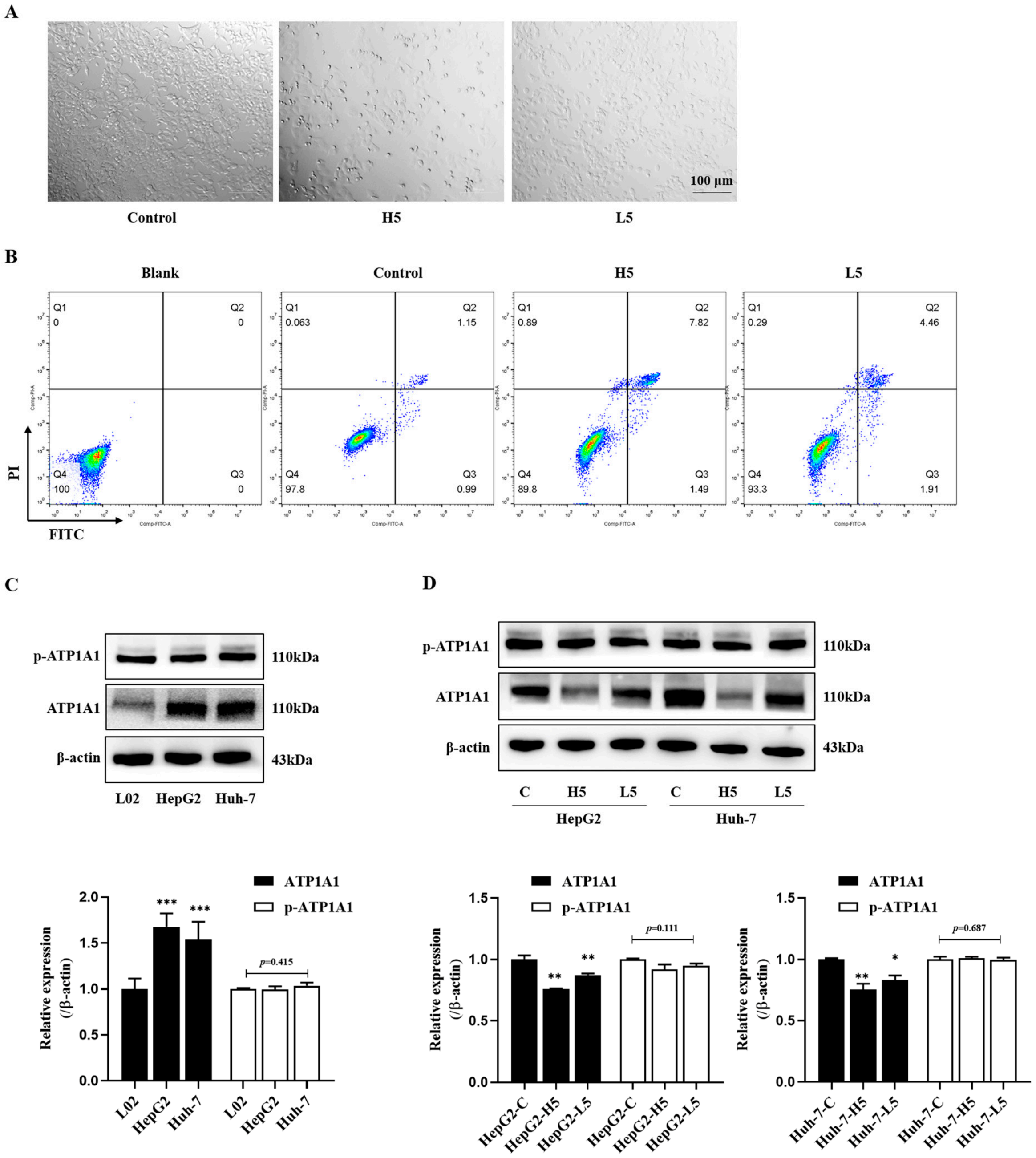

Figure S2 Compound 5 inhibited the proliferation and the level of ATP1A1 protein in Huh-7 cells

L5, low concentration of 5 (12.5 μmol^{-1}); H5, high concentration of 5 (25 μmol^{-1}). (A) The morphology of Huh-7 cells was treated with **5** for 48 hours. (B) Image of cell apoptosis in Huh-7 cells was detected by flow cytometry. The cells were double stained with Annexin V and PI. Early apoptotic cells were stained with Annexin V, and late apoptotic cells were stained with PI. (C) The expression and phosphorylation of ATP1A1 in L02, HepG2 and Huh-7 cells. (D) The effect of **5** on the expression and phosphorylation of ATP1A1 in HepG2 and Huh-7 cells. Data are expressed as the mean ± standard deviation (n=3). $^{*}p$<0.05, $^{**}p$<0.01, $^{***}p$<0.001 *vs.* the control group.

3. The ^{1}H NMR and ^{13}C NMR spectral data of compound 5

^{1}H-NMR ($CDCl_3$, 600 MHz): δ_H 1.56 (3H, s, Me-18), 1.18 (3H, s, Me-19), 1.10 (3H, d, J = 6.3 Hz, Me-21), 7.76 (1H, d, J = 15.9 Hz, H-7'), 6.46 (1H, d, J = 15.9 Hz, H-8'), 1.30 (3H, d, J = 6.2 Hz, Me-6''). ^{13}C-NMR ($CDCl_3$, 150 MHz): δ_C 38.2 (t, C-1), 28.9 (t, C-2), 77.8 (d, C-3), 38.8 (t, C-4), 139.6 (s, C-5), 117.3 (d, C-6), 33.3 (t, C-7), 73.0 (s, C-8), 43.4 (d, C-9), 37.0 (s, C-10), 24.6 (t, C-11), 74.3 (d, C-12), 56.0 (s, C-13), 87.9 (s, C-14), 34.5 (t, C-15), 31.6 (t, C-16), 87.8 (s, C-17), 11.2 (q, C-18), 17.6 (q, C-19), 71.3 (d, C-20), 18.1 (q, C-21), 133.9 (s, C-1'), 128.3 (d, C-2', 6'), 128.9 (d, C-3', 5'), 130.7 (d, C-4'), 146.3 (d, C-7'), 118.4 (d, C-8'), 166.2 (s, C-9'), 95.6 (d, C-1''), 38.6 (t, C-2''), 68.3 (d, C-3''), 73.8 (d, C-4''), 69.2 (d, C-5''), 18.1 (q, C-6'')

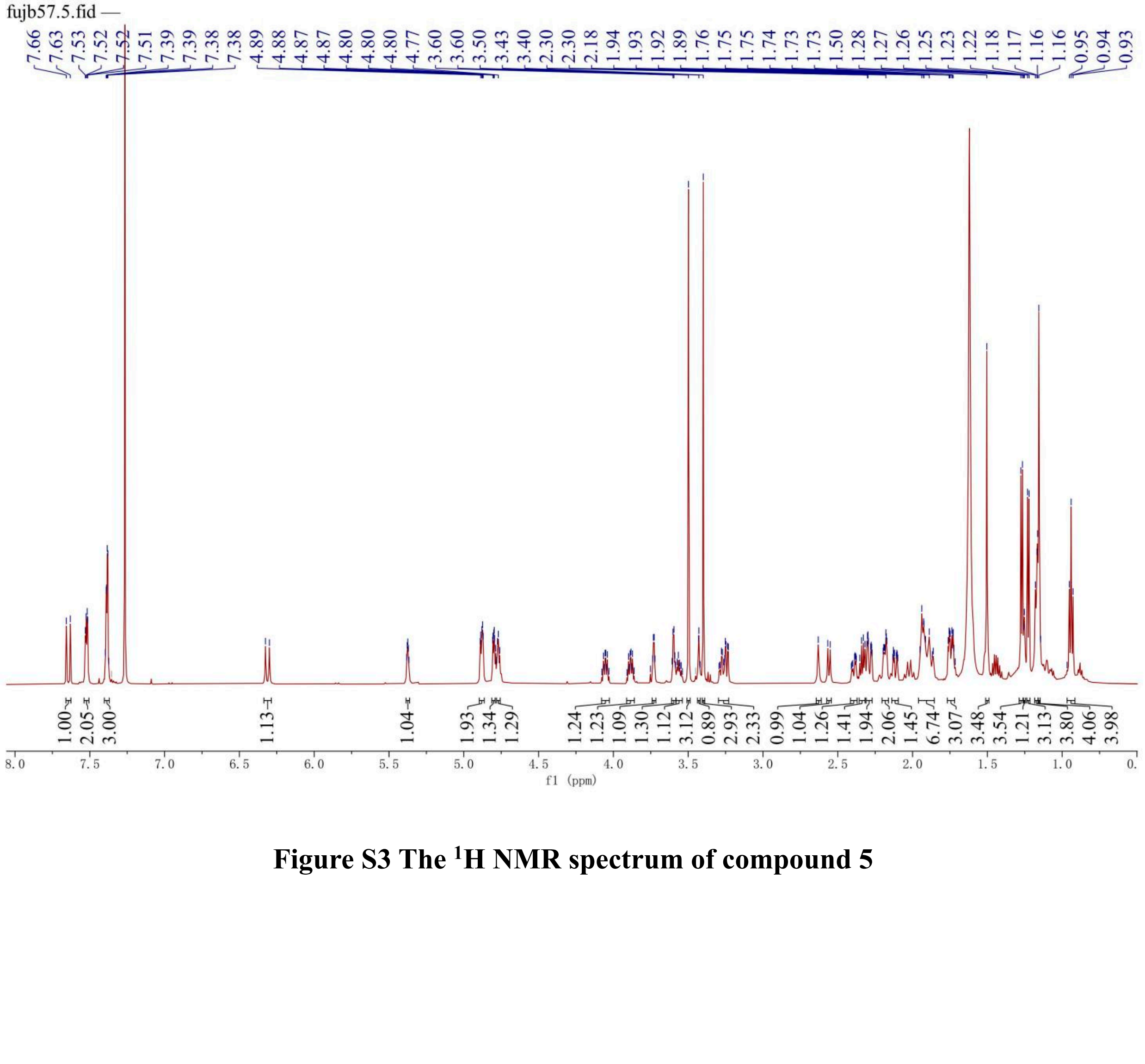

Figure S3 The ^{1}H NMR spectrum of compound 5

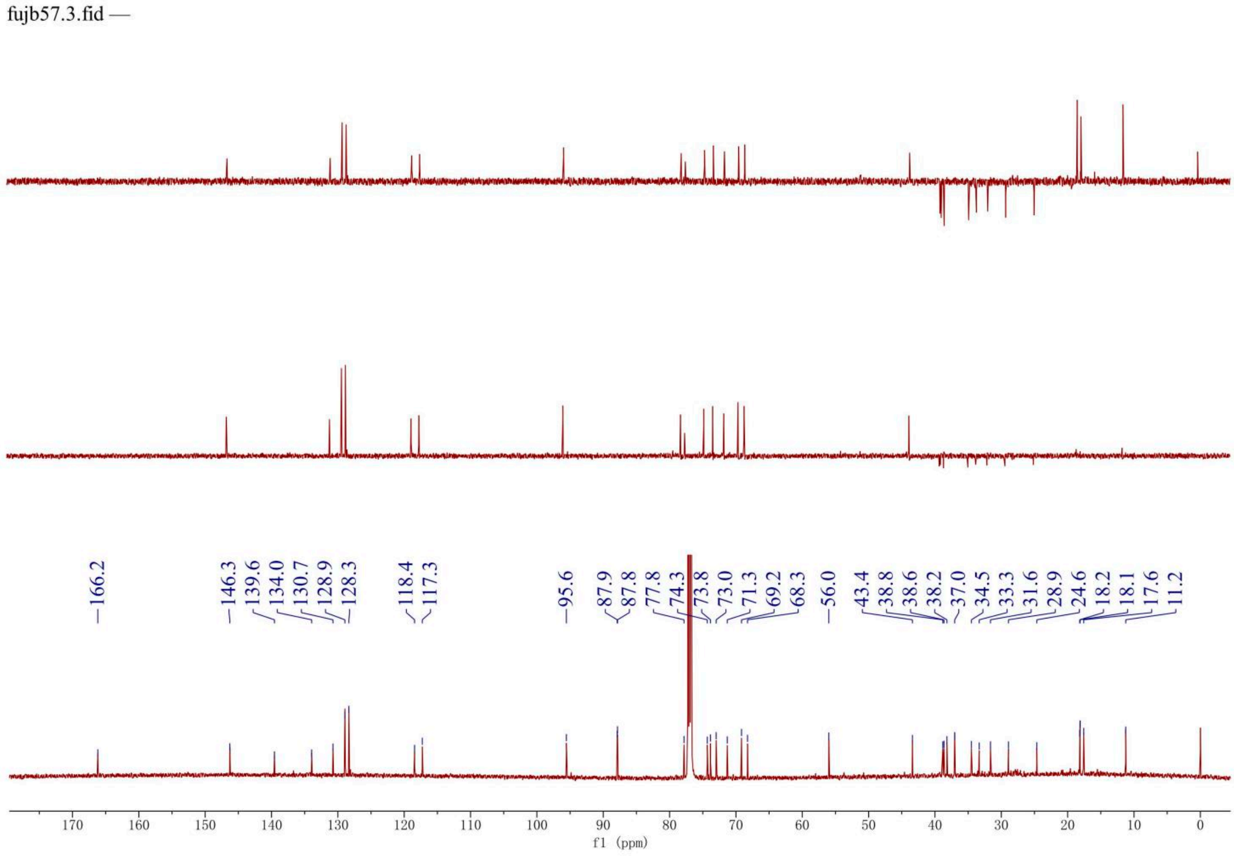

Figure S4 The ^{13}C NMR spectrum of compound 5
